# Optimizing beta cell function through mesenchymal stromal cell‐mediated mitochondria transfer

**DOI:** 10.1002/stem.3134

**Published:** 2020-01-08

**Authors:** Chloe L. Rackham, Ella L. Hubber, Anna Czajka, Afshan N. Malik, Aileen J. F. King, Peter M. Jones

**Affiliations:** ^1^ Department of Diabetes, School of Life Course Sciences, Faculty of Life Sciences and Medicine King's College London London UK

**Keywords:** diabetes, islet transplantation, mesenchymal stromal cells, mitochondrial transfer

## Abstract

Pretransplant islet culture is associated with the loss of islet cell mass and insulin secretory function. Insulin secretion from islet β‐cells is primarily controlled by mitochondrial ATP generation in response to elevations in extracellular glucose. Coculture of islets with mesenchymal stromal cells (MSCs) improves islet insulin secretory function in vitro, which correlates with superior islet graft function in vivo. This study aimed to determine whether the improved islet function is associated with mitochondrial transfer from MSCs to cocultured islets. We have demonstrated mitochondrial transfer from human adipose MSCs to human islet β‐cells in coculture. Fluorescence imaging showed that mitochondrial transfer occurs, at least partially, through tunneling nanotube (TNT)‐like structures. The extent of mitochondrial transfer to clinically relevant human islets was greater than that to experimental mouse islets. Human islets are subjected to more extreme cellular stressors than mouse islets, which may induce “danger signals” for MSCs, initiating the donation of MSC‐derived mitochondria to human islet β‐cells. Our observations of increased MSC‐mediated mitochondria transfer to hypoxia‐exposed mouse islets are consistent with this and suggest that MSCs are most effective in supporting the secretory function of compromised β‐cells. Ensuring optimal MSC‐derived mitochondria transfer in preculture and/or cotransplantation strategies could be used to maximize the therapeutic efficacy of MSCs, thus enabling the more widespread application of clinical islet transplantation.

## Graphical abstract

Human mesenchymal stromal cells (MSCs) transfer their mitochondria to cocultured human islet β‐cells. Fluorescence imaging showed that mitochondrial transfer occurs, at least partially through tunneling nanotube‐like structures. MSC‐mediated mitochondrial donation to islet β‐cells represents a novel mechanism of enhanced islet β‐cell function.

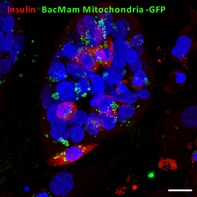

**Significance statement**

Mesenchymal stromal cells (MSCs) have direct effects on islet β‐cells to improve their insulin secretory function. It is well established that the generation of ATP and other metabolic coupling factors by mitochondrial metabolism is essential for nutrient‐induced insulin secretion and that impaired mitochondrial function, and thus reduced oxygen consumption rate, results in defective insulin secretion and reduced islet β‐cell survival. This article reports, for the first time to the authors' knowledge, that human MSCs transfer their mitochondria to cocultured human islet β‐cells. These findings suggest that the mitochondrial donation capacity of MSCs should be harnessed to ensure the functional longevity of transplanted human islets in clinical protocols.

## 1 INTRODUCTION

Allogenic islet transplantation offers the possibility of treating a small subgroup of people with type 1 diabetes (T1D), but the limited availability of human islet material is a major obstacle to the more widespread adoption of islet transplantation as a treatment option for the majority of people with T1D.^1^ Clinical transplantation of allogeneic human islets necessitates a short‐term culture period for safety tests, administration of the transplant recipient to hospital and induction immunotherapy. Unfortunately, the functional viability of islets is compromised by inflammatory, oxidative and hypoxic stresses during this period, with cold ischaemia time and oxygen supply during pancreas procurement contributing to extensive islet cell loss.^2^

Mouse mesenchymal stromal cells (MSCs) derived from multiple tissue sources, including kidney, adipose, and bone marrow (BM), have direct effects on donor islet β‐cells to improve their survival and insulin secretory function during the in vitro culture period prior to transplantation.^3^, ^4^, ^5^, ^6^, ^7^ These in vitro findings correlate with persistent improvements in subsequent islet post‐transplantation function in vivo. Thus, we demonstrated improved graft curative capacity in streptozotocin‐induced diabetic mice transplanted with islets cocultured with MSCs, whether grafted at the experimental renal subcapsular site^4^ or at the clinically preferred intraportal route.^3^ We, and others, have also demonstrated that these findings translate to clinically relevant human islets and human MSCs.^5^, ^8^, ^9^

MSCs can influence islet function through a variety of mechanisms. We have identified MSC‐derived soluble secretory products that mimic some of the beneficial effects of MSCs in vitro,^10^, ^11^ and shown that preculturing islets with a defined cocktail of MSC‐secreted ligands also improved islet graft function in vivo, albeit not to the same extent seen with MSC coculture.^10^ MSC‐derived extracellular matrix further contributes to the beneficial effects of MSCs on islet function,^8^ and a number of studies have highlighted the importance of direct MSC‐islet cell‐cell contact for islet functional survival.^4^, ^6^, ^12^ Studies in other tissues have also demonstrated the capacity of MSCs to act as mitochondria donors by transferring functional mitochondria directly to adjacent cocultured cells in inflammatory and ischemic disease settings, resulting in the rescue of aerobic respiration.^13^, ^14^, ^15^, ^16^, ^17^ Insulin secretion from β‐cells is primarily controlled by mitochondrial ATP generation in response to elevations in extracellular glucose, and islet oxygen consumption rate (OCR) is a key predictor of islet transplantation outcome.^18^, ^19^, ^20^ We have therefore addressed the hypothesis that the MSC‐dependent enhancement of insulin secretory function^3^, ^4^, ^5^, ^6^, ^8^, ^10^, ^12^, ^21^, ^22^ is associated with mitochondrial transfer from MSCs to neighboring islet β‐cells.

## 2 MATERIALS AND METHODS

### 2.1 Human and mouse islet isolation

Human islets were isolated from six nondiabetic donors at the King's College Hospital Islet Transplantation Unit, with appropriate ethical approval (LREC 01‐082). Islets were maintained in CMRL medium supplemented with 2% human albumin, 4 mM glutamine, 2 mM HEPES (pH 7.2‐7.4), and 10 mM nicotinamide at 37°C, 5% CO_2_ prior to establishing human MSC: human islet cocultures, which were maintained in RPMI‐1640 (supplemented with 10% [vol/vol] FCS, 2 mmol/l‐glutamine, and 100 U/mL penicillin/0.1 mg/mL streptomycin). Human islets were handpicked into groups of 80 for culture alone or with MSCs for 1‐3 days, as specified. The characteristics of each donor (age, gender, body mass index [BMI], islet purity, and viability) are specified in Supplementary Table S1. Mouse islets were isolated from male CD1 mice (Charles River, Margate, Kent) aged 8‐12 weeks, by collagenase digestion (1 mg/mL; type XI; Sigma‐Aldrich, Poole, UK) followed by density gradient separation (Histopaque‐1077; Sigma‐Aldrich). After washing with RPMI‐1640 medium, islets were handpicked into groups of 80 for culture alone or with MSCs for 1‐3 days as specified.

### 2.2 Direct contact coculture of islets and MSCs

We used a direct‐contact monolayer configuration to coculture islets with MSCs, as previously described.^3^, ^4^, ^5^, ^8^ Briefly, 100 000 mouse MSCs (Cyagen strain C57BL/6 BM‐derived MSCs or Cyagen strain C57BL/6 Adipose‐derived MSCs; Generon, Slough, UK), or human adipose‐derived MSCs (Stempro Human Adipose‐derived stem cells; Life Technologies Ltd, Paisley, UK) were seeded into Nunclon 35 mm petri dishes, or 35 mm ibitreat ibidi μ‐Dish, high (Thistle Scientific Ltd, Glasgow, UK) for confocal imaging, forming a confluent monolayer of cells within 12 hours. MSCs were cultured in DMEM supplemented with 1% (vol/vol) penicillin/streptomycin solution (Gibco BRL, Gaithersburg, Maryland) supplemented with 10% (vol/vol) FCS and incubated at 37°C in a humidified atmosphere containing 5% CO_2_. The medium was changed after 20 hours, with removal of nonadherent cells. Eighty isolated islets were then added to each petri dish allowing direct cell‐cell contact between the islets and pre‐seeded MSCs. The medium was replaced with RPMI‐1640 (supplemented with 10% [vol/vol] FCS, 2 mmol/l‐glutamine, and 100 U/mL penicillin/0.1 mg/mL streptomycin, Sigma‐Aldrich).

### 2.3 Labeling of MSC‐derived mitochondria

Human and mouse MSCs were transduced with Cell Light BacMam Mitochondria‐GFP (Life Technologies Ltd), according to manufacturer's instructions. Briefly, MSCs were trypsinized and seeded at a density 100 000 cells (unless otherwise specified) per 35 mm ibitreat ibidi μ‐Dish, high (Thistle Scientific Ltd, Glasgow, UK) and BacMam Mitochondria‐GFP (Life Technologies Ltd) added to the MSC media (DMEM). BacMam mitochondria‐GFP is targeted to the mitochondrial matrix pyruvate dehydrogenase enzyme complex, ensuring fluorescence labeling of MSC‐derived mitochondria. After 20 hours, the BacMam reagent was removed, MSCs washed three times in RPMI and expression of the GFP‐transgene confirmed by fluorescence microscopy, prior to setting up MSC‐islet cocultures, as described above.^3^, ^4^, ^5^, ^8^

### 2.4 Immunostaining of islet β‐cells and tunneling nanotubes

Intact MSC: islet cocultures were immunostained with insulin antibodies for detection of islet β‐cells. Briefly, islet‐MSC cocultures were washed in PBS, fixed in 3.7% (vol/vol) formalin (Sigma‐Aldrich) or 2% paraformaldehyde where phalloidin staining was included, for 25 minutes, before permeabilization with 0.3% Triton X‐100 (Sigma‐Aldrich). Cocultures were incubated for 1 hour at 37°C with a polyclonal guinea pig anti‐insulin antibody (1:100, Dako, Ely, UK) or monoclonal mouse anti‐insulin antibody (1:100, Sigma). Tunneling nanotubes (TNTs) were visualized by staining F‐actin with phalloidin (647) and fixed cocultures washed in PBS prior to incubation for 1 hour at 37°C with an Alexa Fluor594‐conjugated donkey anti‐guinea pig or anti‐mouse secondary antibody and a goat polyclonal anti‐GFP (FITC) secondary antibody (1:100, Abcam, Cambridge, UK). Cell nuclei were stained with 4′,6‐diamidino‐2‐phenylindole (DAPI). Imaging of intact MSC cocultures was performed using confocal laser scanning microscopy (Nikon A1 inverted). Imaging was started at the MSC‐islet interface and upward through the first 2‐3 layers of islet cells. Confocal Z‐stack projected images composed of 10‐25× 0.88 μm slices (as specified for each micrograph) were produced to investigate interactions between MSCs and cocultured islets. The individual 0.88 μm slices were also analyzed to confirm the intracellular localization of MSC‐derived mitochondria to islet cells. Twenty‐four to 33 individual islets were imaged per group and analyzed semiquantitatively by a blinded investigator, using FIJI software (https://fiji.sc/). Specifically, images were stacked, a threshold set to remove background fluorescence, and the percentage of remaining GFP signal within the selected islet area was calculated.

### 2.5 Islet mitochondrial bioenergetics

The Seahorse extracellular flux analyzer XF24 (Agilent, Cheshire, UK) was used to measure islet OCR, as per manufacturer's instructions. Briefly, islets which had been cultured alone or with MSCs^3^, ^4^, ^5^, ^8^ were washed in XA basal media (Agilent) supplemented with 2 mM glucose and 1% FBS, before hand‐picking into groups of 100 islets/500 μL XA basal media, per XF24 well. Islet screens were carefully added to enclose islets in the depression of the islet microplate. OCR was measured under basal (2 mM) and maximal (20 mM) glucose concentrations, as well as with drugs acting on the respiratory chain: oligomycin (ATP synthase inhibitor; 10 μM, Sigma) and FCCP (uncoupler; 1 μM, Sigma).^23^ Data were normalized to initial OCR under basal conditions to account for variations in islet size and are reported as percentage of basal OCR. Glucose‐stimulated respiration was calculated by dividing the first OCR measurement after injection of 20 mM glucose by the last basal OCR measurement and multiplying by 100.

### 2.6 Islet insulin secretory function

Insulin secretion in vitro was assessed in static incubations of isolated islets. Islets were preincubated for 2 hours in RPMI containing 2 mM glucose. Groups of three islets were transferred into 1.5 mL Eppendorf tubes and incubated at 37°C in a bicarbonate‐buffered physiological salt solution, containing 2 mM CaCl^2^ and 0.5 mg/mL BSA and either 2‐ or 20‐mM glucose. Samples of the incubation medium were taken after 1 hour and stored at −20°C until assayed for insulin content using in‐house radioimmunoassay.^11^, ^24^

### 2.7 Statistical analysis

Results are expressed as means ± SEM. ANOVA with Bonferroni's multiple comparison post hoc test was used for comparisons among multiple groups. A Student's *t* test for comparisons between two groups was used. A *P* value of .05 was considered significant. All statistical analysis was performed using GraphPad Prism version 6.

## 3 RESULTS

### 3.1 Islet mitochondrial bioenergetics after MSC coculture

**Figure 1:**
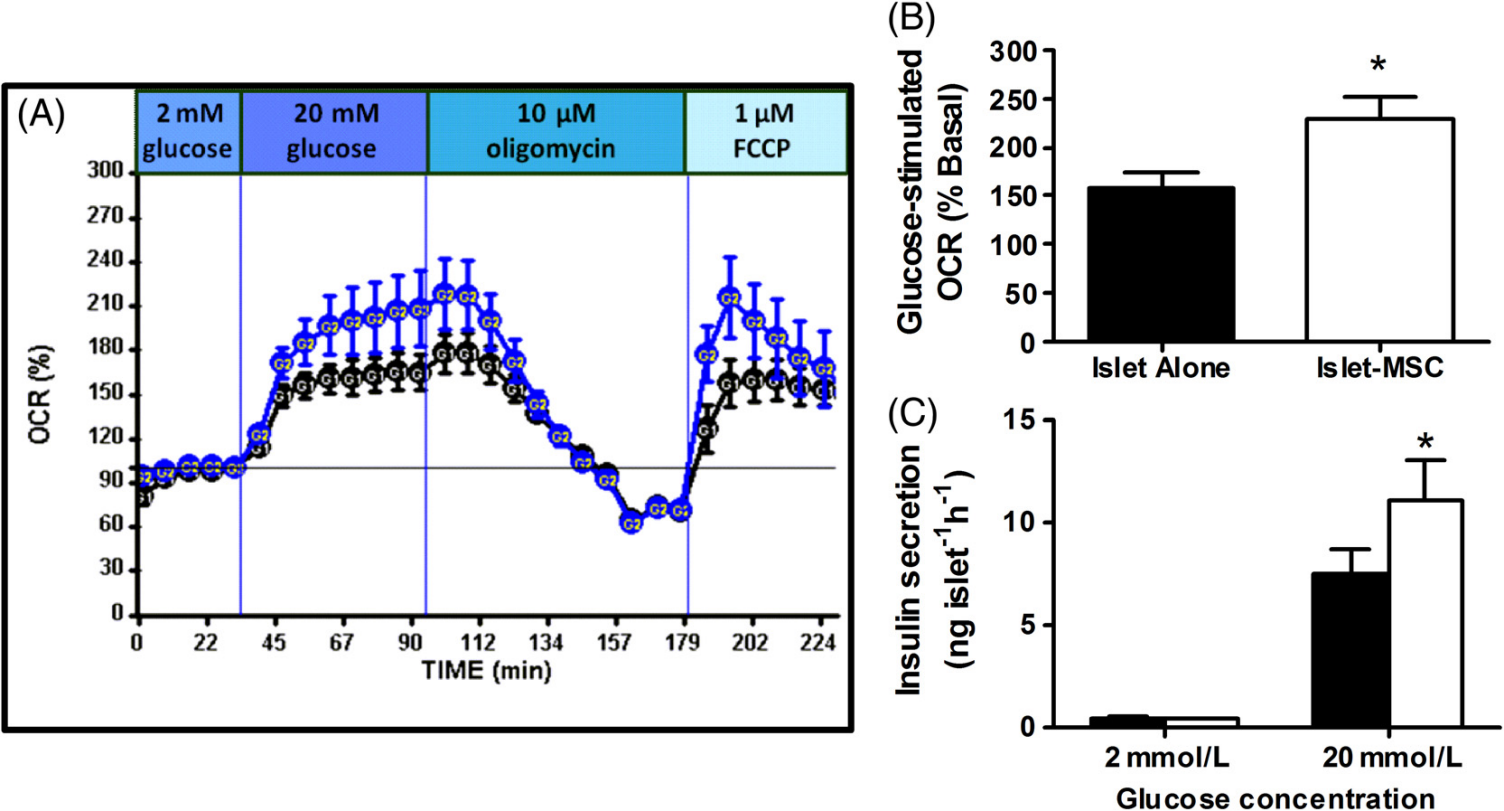
Islet mitochondrial bioenergetics after MSC coculture. A, Oxygen consumption rate (OCR) of mouse islets precultured alone (black circles) or with mouse adipose MSCs (blue circles), measured using the seahorse XF24 analyzer. OCR was measured under basal (2 mM) and maximal (20 mM) glucose concentrations, as well as with drugs acting on the respiratory chain: oligomycin (ATP synthase inhibitor; 1 μM, Sigma) and FCCP (uncoupler; 10 μM, Sigma). OCR was measured using 100 islets per well (n = 8 wells per group; results are representative of three separate coculture experiments). B, Glucose‐stimulated OCR is increased in MSC cocultured islets, 100 islets per well (n = 8 wells per group), *P < .05 vs islets precultured alone, Student's *t* test. C, Insulin release at 2 and 20 mmol/L glucose of 10 replicates of triplicate islets cultured for 3 days with mouse adipose MSCs (white bars) or without MSCs (black bars), *P < .05 vs absence of MSCs at the same glucose concentration (two‐way ANOVA with Bonferroni post hoc test). MSC, mesenchymal stromal cells, OCR, oxygen consumption rate

The generation of ATP and other metabolic coupling factors by mitochondrial metabolism is essential for nutrient‐induced insulin secretion^25^ and glucose‐stimulated OCR is an important predictor of islet transplantation outcomes.^18^, ^19^, ^20^ To determine whether MSCs induce alterations in islet mitochondrial bioenergetics, we measured mouse islet OCR using the seahorse XF24 islet respirometry platform. Islets that had been cocultured with MSCs were separated from the MSC monolayer, by gentle pipetting, prior to measurements of islet OCR and glucose‐stimulated insulin secretion (GSIS). Our measurements of islet oxygen consumption demonstrate improved islet mitochondrial bioenergetics in MSC cocultured islets (Figure 1A). After a 2‐hour preincubation in low glucose (2 mM), control islets stimulated with 20 mM glucose demonstrated a clear increase in OCR to approximately 1.6‐fold their basal level. In MSC cocultured islets, glucose‐stimulated OCR was increased to twofold of the basal level (Figure 1B). Upon addition of 10 μM oligomycin (an ATP synthase inhibitor), respiration was reduced in both control and MSC cocultured islets. Addition of 1 μM FCCP, which induces maximal respiration by uncoupling oxidative phosphorylation from the electron transport chain, caused a sharp increase in OCR which was more pronounced in MSC cocultured islets than in control islets. The concentrations of glucose used for basal and glucose‐stimulated OCR measurements mirror those used for our standard static islet insulin secretion assays (Figure 1C). As shown in Figure 1C, we consistently observe an MSC‐dependent potentiation of GSIS in both mouse^3^, ^4^ and human islets,^5^, ^8^ and using MSCs derived from multiple tissues including adipose, BM, and kidney.^3^, ^4^, ^5^, ^8^ We now demonstrate that the MSC‐mediated improvements in islet insulin secretory function are associated with improved islet mitochondrial bioenergetics.

### 3.2 Mitochondrial transfer from human MSCs to human islet β‐cells

**Figure 2:**
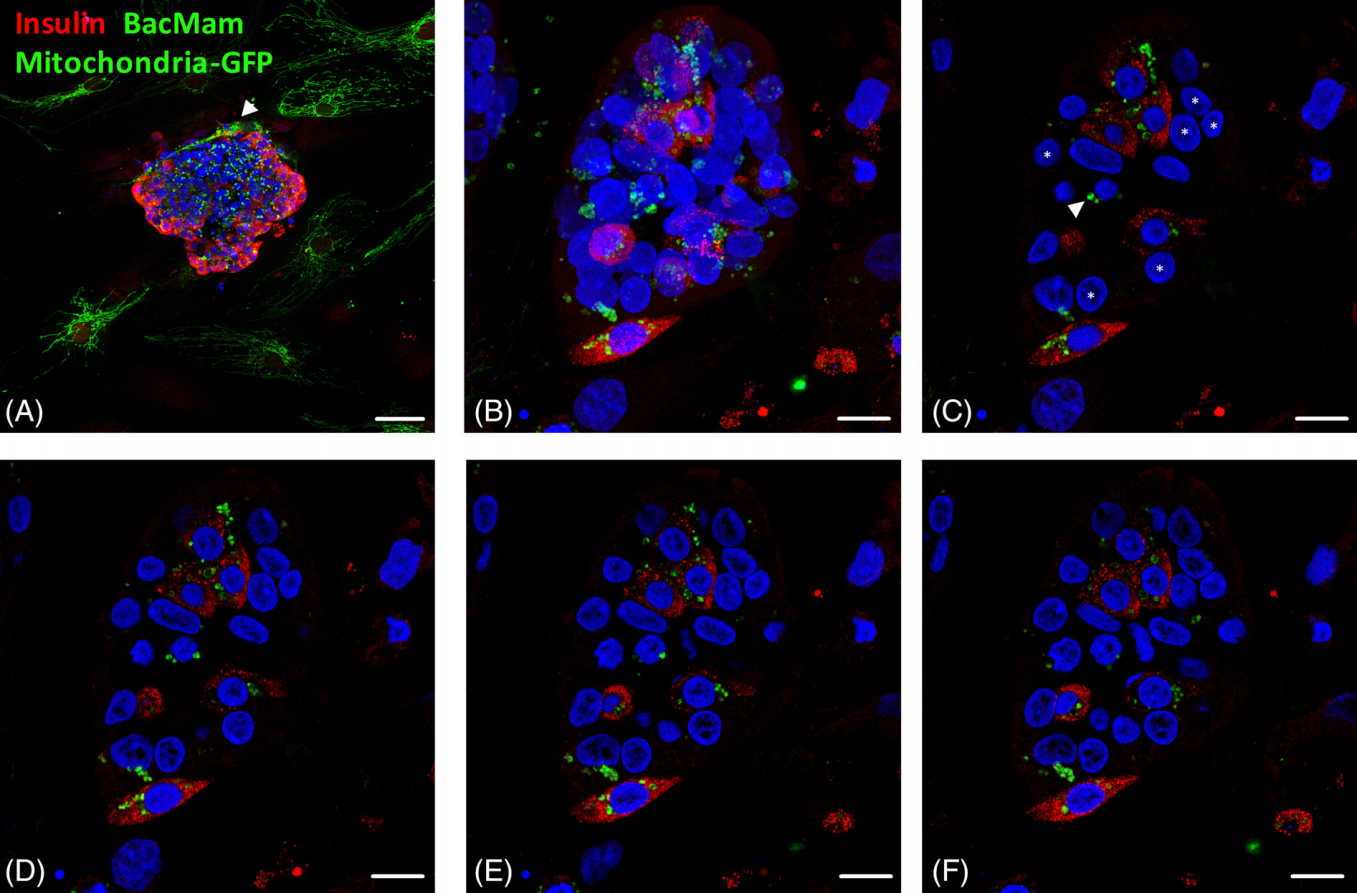
Mitochondrial transfer from human mesenchymal stromal cells (MSCs) to human islet beta cells. Confocal micrographs showing representative images of human adipose MSC cocultured human islets. Green indicates MSC‐derived BacMam mitochondria‐GFP labeling, which was clearly abundant in the majority of insulin‐immunoreactive (red) β‐cells. Blue represents DAPI. A, Human adipose MSCs (P5) were seeded and transduced with BacMam mitochondria‐GFP at a density of 50 000 MSCs per 35 mm dish and cocultured with human islets for 1 day before immunostaining with insulin antibodies, ×20 magnification; scale bar = 50 μm. B‐F, Confocal micrographs showing representative images of 2‐day human adipose (P5) MSC cocultured human islets. B, Composite Z‐projection of 25× 0.88 μm optical sections of a series of consecutive human islet slices starting at the MSC: islet interface and upward. C‐F, A series of individual consecutive 0.88 μm insulin immunostained human islet slices starting at the MSC: islet interface and upward, indicating the intra β‐cell localization of MSC‐derived BacMam mitochondria‐GFP. Scale bar = 10 μm. Images are representative of six separate human MSC: human islet coculture experiments

To assess whether MSCs transfer mitochondria to β‐cells in cocultured islets, we fluorescently tagged the mitochondria in human adipose‐MSCs by transduction with BacMam mitochondria‐GFP 1 day prior to direct contact coculture with human islets. The mitochondrial networks of MSCs were visualized by fluorescence microscopy, demonstrating MSCs adhering to the outside perimeter of islets after 1 day of coculture (Figure 2A, arrowhead). Mitochondria‐GFP particles or vesicles, termed hereafter “microvesicles,” were localized to the islet and rarely associated with surrounding MSCs that were not in close proximity to neighboring islets. After a 48‐hour coculture period, analysis of three‐dimensional (3D) Z‐projections of 25× 0.88 μm optical slices (Figure 2B), from the MSC‐islet interface and upward through the first 2‐3 layers of islet cells, revealed extensive MSC‐derived mitochondrial transfer to human islet β‐cells. Mitochondria‐GFP microvesicles, as well as more diffuse MSC‐derived mitochondrial‐GFP labeling, were clearly evident in the majority of β‐cells in all islets. A series of consecutive 0.88 μm optical slices (Figure 2C‐F) demonstrate the intra‐β‐cell localization of the mitochondrial‐GFP. Islet non‐β‐cells were also recipient to MSC‐derived mitochondria (Figure 2C, arrowhead); however, it was clear that the majority of non‐β cells, presumably mainly alpha‐, delta‐, or endothelial‐cells, within the islet structure were not labeled with Mitochondria‐GFP (Figure 2C, asterisks). These observations are consistent with the physical transfer of MSC‐derived mitochondria to human islet β‐cells.

### 3.3 Time course of mitochondrial transfer from human MSCs to human islet β‐cells

**Figure 3:**
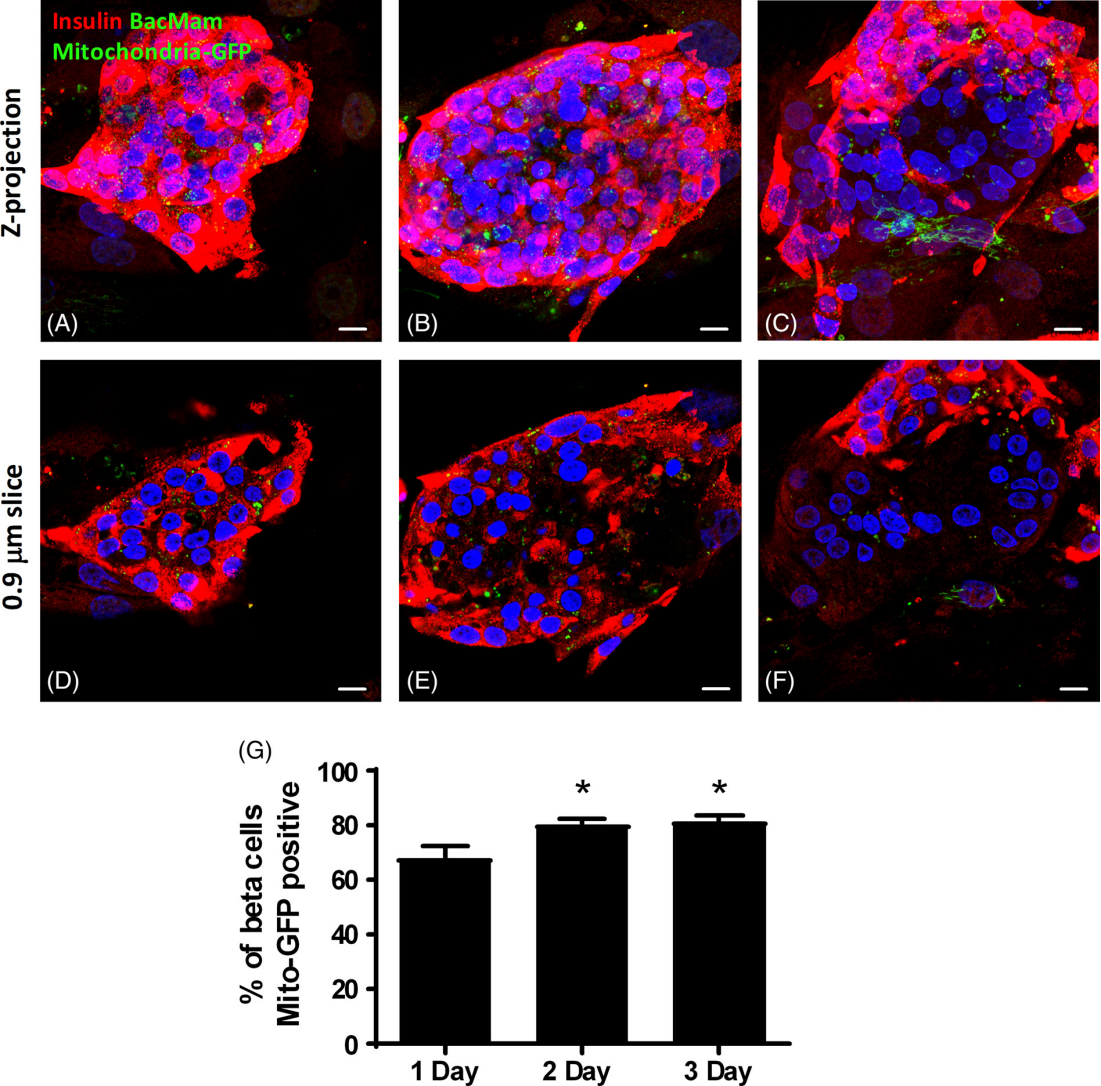
Time course of mitochondrial transfer from human mesenchymal stromal cells (MSCs) to human islet beta cells. A‐C, Composite Z‐projections of 25× 0.88 μm optical sections of a series of consecutive human islet slices starting at the MSC: islet interface and upward. D‐F, Individual 0.88 μm insulin immunostained human islet slices (within the first layer [10 μm] of islet cells), indicating the intra β‐cell localization of MSC‐derived BacMam mitochondria‐GFP. Confocal micrographs are representative images of 1‐ (A, D), 2‐ (B, E), and 3‐day (C, F) human adipose MSC cocultured human islets. The percentage of human islet β‐cells (insulin immunoreactive: red) that are mitochondria‐GFP positive was quantified at each time point. Blue represents DAPI. Green indicates MSC‐derived BacMam mitochondria‐GFP labeling. Magnification ×60, scale bar = 10 μm. G, Quantification of the percentage of β‐cells recipient to MSC‐derived mitochondria in 0.88 μm slices 5, 10, 15, 20, and 25 μm from the MSC: islet interface after 1, 2, and 3 days of coculture, *P < .05 vs 1‐day MSC cocultured islets, one‐way ANOVA with Bonferroni's post hoc test

To determine the time course and extent of mitochondrial transfer from human adipose‐MSCs to cocultured human islets, we quantified the percentage of islet β‐cells that were mitochondria‐GFP positive after 1 (Figure 3A), 2 (Figure 3B), and 3 days (Figure 3C) of coculture. Mitochondria‐GFP could be visualized for at least 25 μm from the MSC: islet interface into the outer 2‐3 layers of islet cells. In some islets, mitochondria‐GFP was evident for at least 40 μm into the 3D islet architecture. We quantified the percentage of β‐cells recipient to MSC‐derived fluorescent mitochondria in individual 0.88 μm slices (Figure 3D‐F) 5, 10, 15, 20, and 25 μm from the MSC: islet interface after 1, 2, and 3 days of coculture. The percentage of human islet β‐cells that were mitochondria‐GFP positive after 1 day of coculture was variable, ranging from 28.6% to 100%, with a mean of 68.2% ± 4.3% of β‐cells containing MSC‐derived fluorescent mitochondria. The percentage of human islet β‐cells positive for mitochondria‐GFP after 2 and 3 days of coculture was less variable (57.1%‐100% and 58.3%‐100%, respectively) and the mean percentage of β‐cells containing MSC‐derived fluorescent mitochondria increased to 80.9% ± 2.1% and 81.3% ± 2.1% in 2‐ and 3‐day cocultured islets, respectively, as shown in Figure 3G.

### 3.4 Mechanism of mitochondrial transfer from human MSCs to human islets

**Figure 4:**
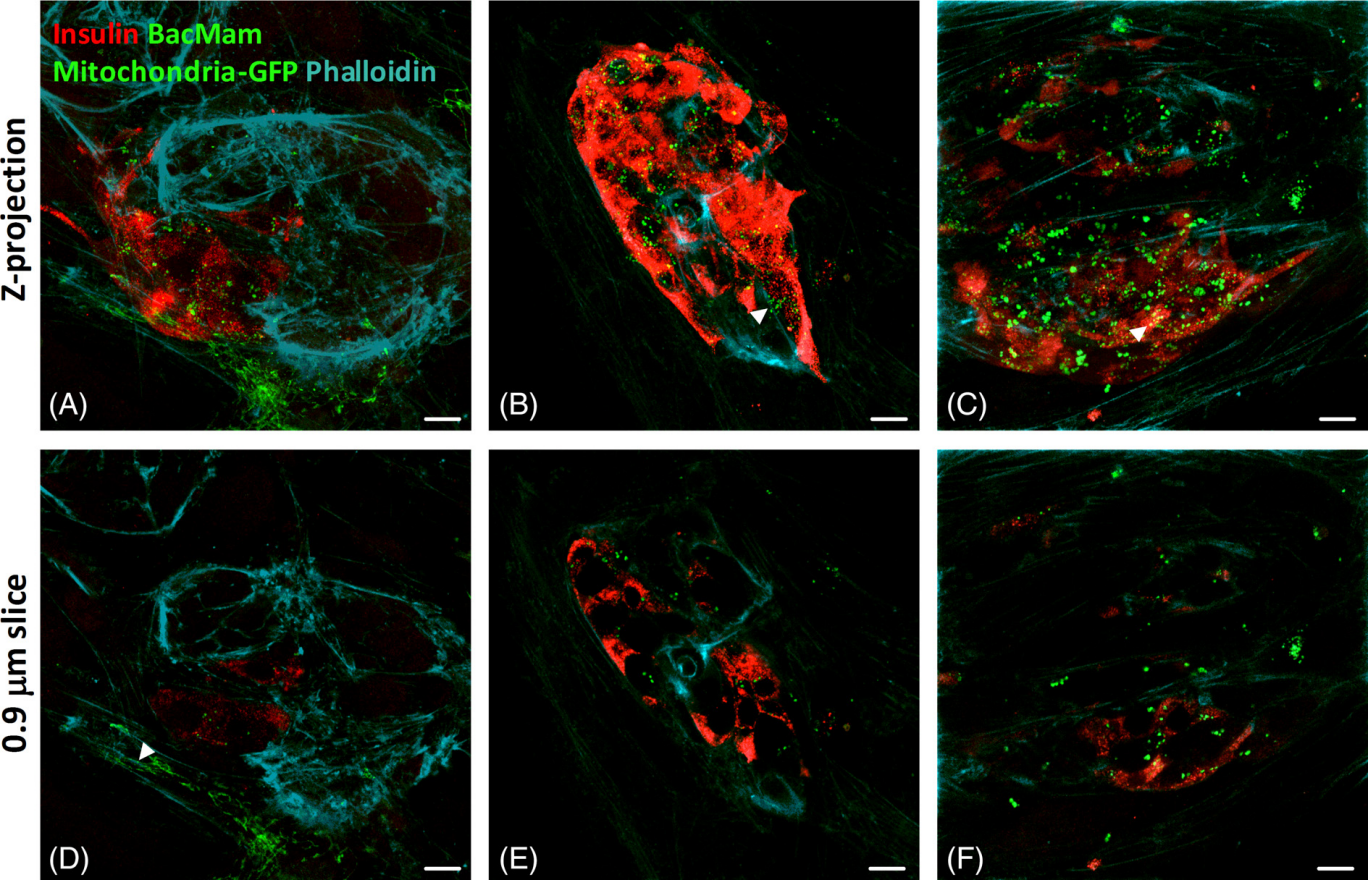
Mechanism of mitochondrial transfer from human mesenchymal stromal cells (MSCs) to human islets. A‐C, Composite Z‐projections of 20× 0.88 μm optical sections of a series of consecutive human islet slices starting at the MSC: islet interface and upward. D‐F, Individual 0.88 μm human islet slice, within the first layer (10 μm) of islet cells. Confocal micrographs show representative images of 1‐ (A, D), 2‐ (B, E), and 3‐day (C, F) human adipose (P5) MSC cocultured human islets. Green indicates MSC‐derived BacMam mitochondria‐GFP labeling. Red represents insulin immunostained β‐cells. Cyan represents phalloidin staining of F‐actin. Blue represents DAPI. Magnification ×60, scale bar= 10 μm

A number of mechanisms of mitochondrial transfer from MSCs to recipient cells have been proposed, including TNTs^14^, ^15^, ^26^ and extracellular vesicles (EVs).^15^, ^27^, ^28^ TNTs are cytoskeletal‐derived ultrafine structures constituted of F‐actin,^29^ as observed with Phalloidin staining.^14^ Filopodia‐like protrusions constituted of F‐actin could be visualized extending from the MSC cell body toward islets in direct contact coculture. We found evidence of these TNT‐like structures between human MSCs and human islets after 1 (Figure 4A), 2 (Figure 4B), and 3 days (Figure 4C) of coculture. At all time points, MSC‐derived mitochondrial‐GFP microvesicles were prevalent both adjacent to (Figure 4B, arrowhead) and independent of these actin‐based structures (Figure 4C, arrowhead). The actin cytoskeleton of the islet cells was also clearly visible at all time points, which complicated visualization of fluorescent mitochondria transfer through the actin‐derived TNTs. However, analysis of individual 0.88 μm slices at each time point (Figure 4D‐F) revealed images consistent with mitochondrial transfer through MSC‐derived TNTs to neighboring human islet cells (Figure 4D, arrowhead). Lower magnification images (Figure 2A) revealed that fluorescent mitochondria‐GFP microvesicles were primarily observed where MSCs were in direct contact with β‐cells, suggesting that MSC‐islet cell adhesion is required for the formation of microvesicles and transfer of MSC‐derived mitochondria to neighboring β‐cells. The abundance of mitochondria‐GFP microvesicles, observed using six separate human islet preparations, suggest that MSC‐derived EVs are another important mechanism of mitochondrial transfer from MSCs to cocultured human islet β‐cells.

### 3.5 Mitochondrial transfer is more extensive to human islets than to mouse islets

**Figure 5:**
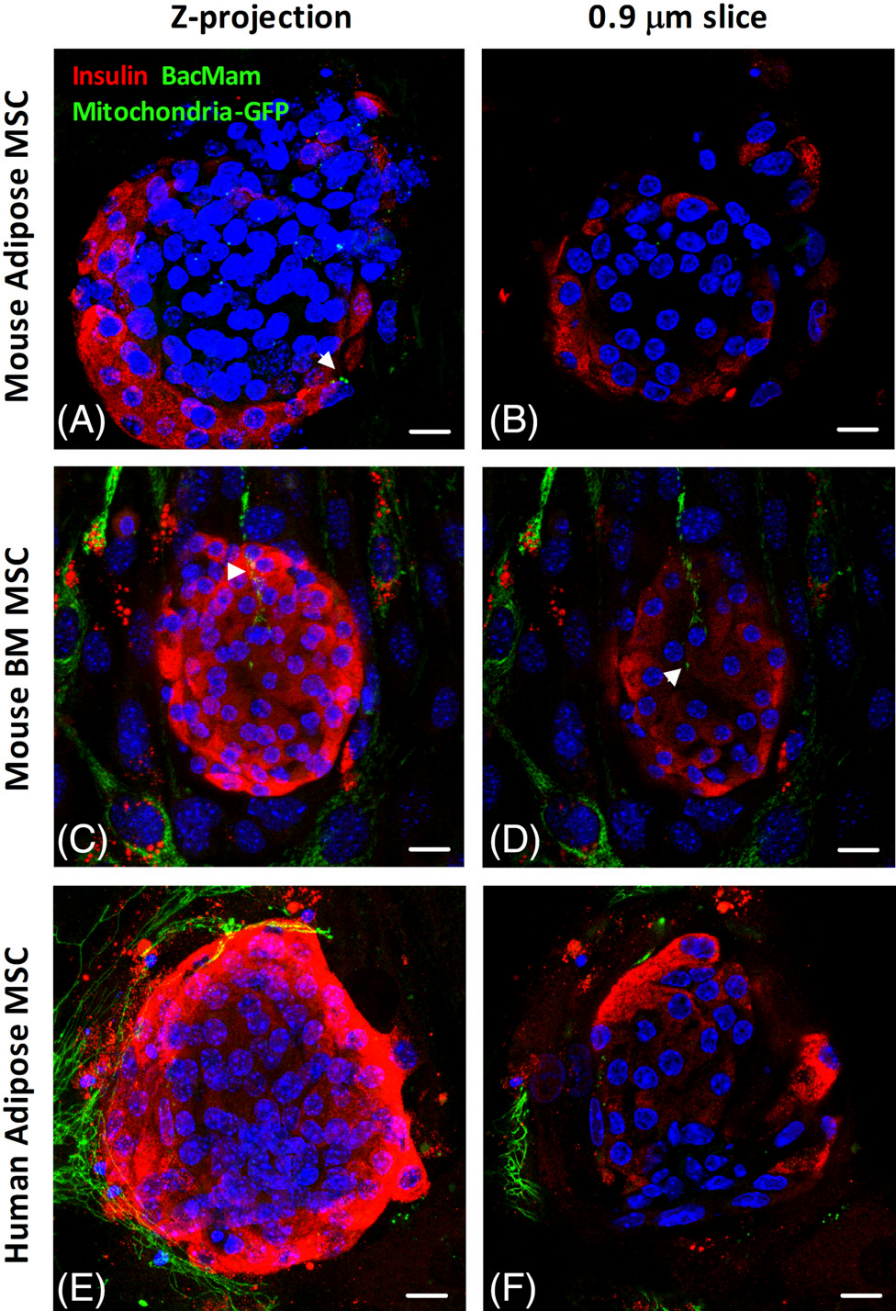
Mitochondrial transfer is more extensive to human islets than to mouse islets. Confocal micrographs showing representative images of mesenchymal stromal cells (MSCs) transduced with BacMam mitochondria‐GFP, seeded at a density of 100 000 MSCs per 35 mm Petri dish, prior to coculture with mouse islets. A,B, P8 mouse adipose MSC 2‐day cocultured mouse islets. Green indicates diffuse MSC‐derived BacMam mitochondria‐GFP labeling in a composite Z‐projection of 20× 0.88 μm optical sections of a series of consecutive mouse islet slices starting at the MSC: islet interface and upward (A). B, A single 0.88 μm optical section within the outer layer (first 10 μm) of mouse islet cells. A,B, Magnification ×60, scale bar = 10 μm. C,D, Green indicates mouse bone marrow (BM) (P8) MSC‐derived BacMam mitochondria‐GFP labeling, demonstrating evidence of mitochondrial transfer through mouse BM MSC‐derived cytoplasmic extensions into the outer layer of mouse islets, with more diffuse mitochondria‐GFP labeling in the surrounding insulin‐immunoreactive (red) β‐cells also evident. Blue represents DAPI. C, Composite Z‐projection of 10× 0.88 μm optical sections of a series of consecutive mouse islet slices starting at the MSC: islet interface and upward. D, A single 0.88 μm optical section within the outer layer (first 10 μm) of mouse islet cells. C,D, Magnification ×20, scale bar = 20 μm. E,F, Green indicates human adipose (P5) MSC‐derived BacMam mitochondria‐GFP labeling in a composite Z‐projection of 25× 0.88 μm optical sections (E) of a series of consecutive mouse islet slices starting at the MSC: islet interface and upward. F, A single 0.88 μm optical section within the outer layer (first 10 μm) of mouse islet cells, demonstrating less extensive MSC‐derived mitochondrial transfer to mouse insulin‐immunoreactive (red) β‐cells compared with that in human islet β‐cells (as represented in Figures 2, 3, 4). E,F, Magnification ×60, scale bar = 10 μm. These experiments were replicated with three separate mouse islet isolations

We next sought to determine whether mitochondrial transfer occurs between mouse MSCs and cocultured mouse islets which are more accessible for experimental investigation. After 2 days, we observed diffuse MSC‐derived mitochondrial‐GFP labeling (Figure 5A, arrowhead) within mouse islets cultured in direct contact with mouse adipose MSCs (Figure 5A,B). However, the defined intra‐β‐cell localization and abundance of vesicular mitochondrial‐GFP labeling which we consistently observed in human adipose MSC cocultured human islets (Figures 2, 3, 4) was not evident in mouse islets. Heterogeneity in mitochondrial transfer capacity between different MSC tissue sources have been reported,^30^ so we also investigated the potential for mouse BM‐MSCs to transfer GFP‐labeled mitochondria to neighboring mouse islet cells. After a 3‐day coculture period, we visualized mitochondrial transfer to only a small number of islet cells with mitochondria‐GFP labeling being localized to MSC‐derived cytoplasmic extensions into the outer layer of islet cells at the MSC‐islet interface, as shown in composite Z‐projection micrographs (Figure 5C, arrowhead) and individual 0.88 μm optical slices (Figure 5D). More diffuse mitochondria‐GFP labeling was also visualized in proximity to the MSC‐derived cytoplasmic extensions (Figure 5D, arrowhead). To determine whether human MSCs are more effective mitochondria donors than mouse MSCs we cocultured mouse islets with mitochondrial GFP‐labeled human adipose MSCs. 3D Z‐projections of 25× 0.88 μm optical slices starting at the MSC‐islet interface and upward through the outer 2‐3 layers of mouse islet cells (Figure 5E), as well as analysis of individual 0.88 μm slices (Figure 5F) did not reveal extensive mitochondrial transfer from human adipose MSCs to mouse islets. Thus, the lack of extensive mitochondria transfer to mouse β‐cells is unlikely to be due to the mitochondrial donation capacity of MSCs, but most likely reflects differences between the ability of mouse and human β‐cells to act as mitochondrial recipients.

### 3.6 Enhanced mitochondrial transfer to hypoxia‐exposed mouse islets

**Figure 6:**
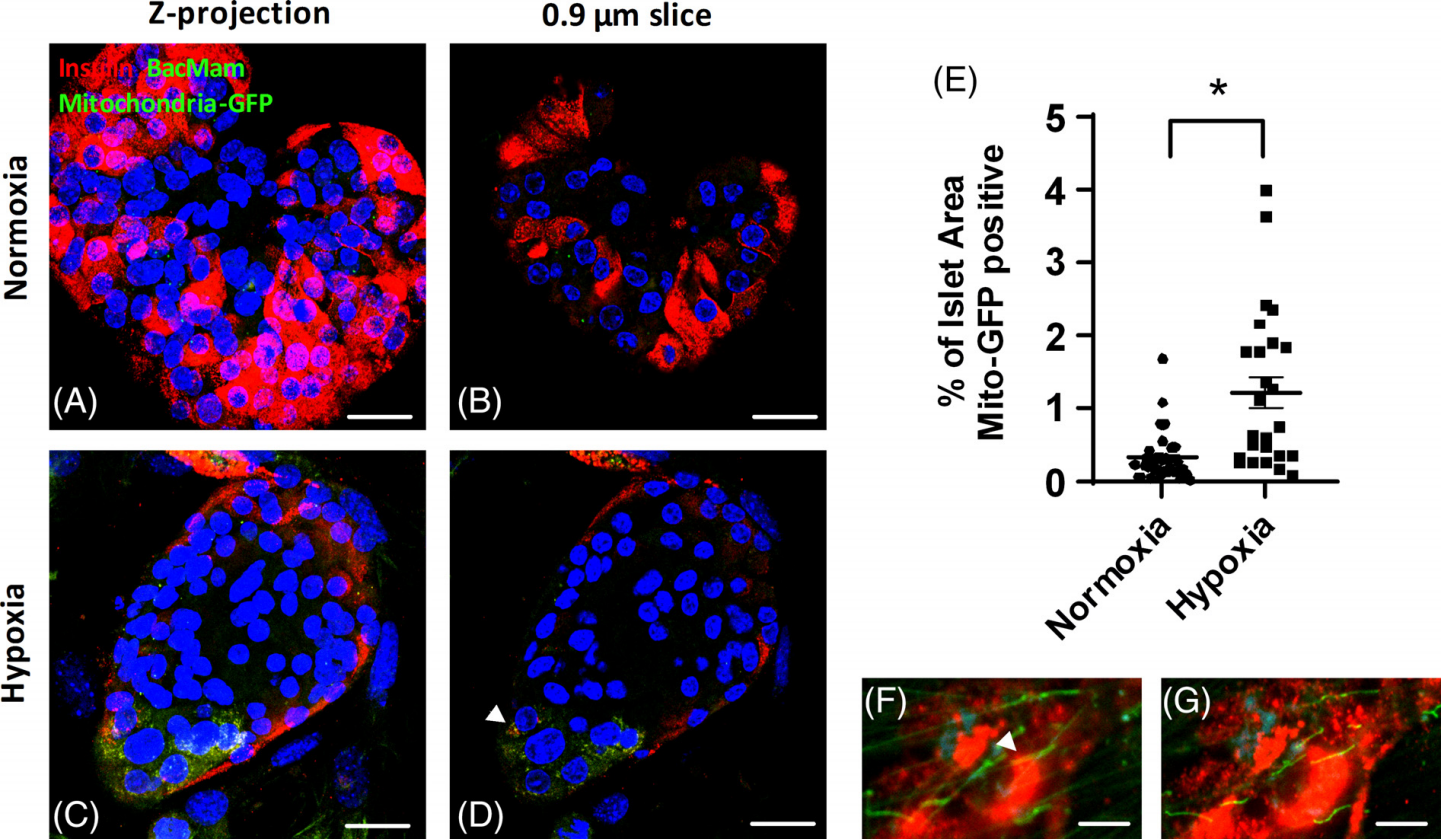
Enhanced mitochondrial transfer to hypoxia‐exposed mouse islets. Confocal micrographs showing representative 3‐day cocultured mouse islets, which were exposed to hypoxia (1% O_2_) for 16 hours before coculturing with mesenchymal stromal cells (MSCs). Green indicates MSC‐derived BacMam mitochondria‐GFP labeling, and red indicates insulin immunostaining of β‐cells and blue represents DAPI (A‐D and F‐G). Cyan represents phalloidin (F,G) staining of F‐actin, indicative of TNTs. A,C, Composite Z‐projection micrographs of 25× 0.88 μm optical sections of a series of consecutive mouse islet slices starting at the MSC: islet interface and upward, in control mouse islets cultured under normoxia (A) and hypoxia pre‐exposed islets (C), subsequently cocultured with mouse bone marrow (BM) MSCs. Magnification ×60, scale bars = 10 μm. B,D, Individual 0.88 μm insulin immunostained mouse islet slices, within the outer layer (first 10 μm) of cells of islets cultured under normoxia (B) and hypoxia pre‐exposed islets (D). Magnification ×60, scale bars = 25 μm. E, Semiquantitative analysis of mitochondrial transfer determining the percentage of each islet area occupied by BacMam mitochondrial‐GFP labeling in 24‐33 separate islets, *P < .001 vs normoxia cultured islets, Student's *t* test. Data presented are representative of three independent experiments. F,G, Human adipose MSC cocultured mouse islets pre‐exposed to hypoxia. Phalloidin staining of F‐actin in a composite Z‐projection of 25× 0.88 μm optical sections of a series of consecutive insulin immunostained mouse islet slices (F) and an individual 0.88 μm slice (G) demonstrating BacMam mitochondrial GFP‐labeling within F‐actin based TNT‐like ultrastructures. Magnification ×60, scale bars = 5 μm

Reports of mitochondria transfer in other tissues are often in models of ischemic or inflammatory disease,^13^, ^14^, ^15^, ^16^, ^17^ consistent with recipient cells responding to cellular stressors by signaling to MSCs to initiate mitochondrial transfer. Isolated human islets are less robust than mouse islets and express a hypoxic molecular signature during the in vitro culture period prior to transplantation,^31^ which is not seen in isolated mouse islets. To determine whether hypoxia influences the extent of mitochondrial transfer from MSCs to islets, we exposed mouse islets to hypoxia (1% oxygen) for 16 hours prior to coculture with mouse or human MSCs. Control mouse islets cultured under normoxic conditions (20% oxygen) demonstrated diffuse mitochondrial‐GFP labeling (Figure 6A,B), as shown previously in Figure 5. In contrast, the extent of mitochondrial transfer to hypoxic mouse islets was more extensive, as shown in 3D Z‐projection micrographs (Figure 6C). The insulin immunostaining intensity was notably weaker in hypoxic mouse islets, as expected, but 0.88 μm islet slices (Figure 6D) confirmed the intracellular localization of MSC‐derived mitochondria within islet cells, including insulin‐positive β‐cells (Figure 6D, arrowhead). Semiquantitative analysis of mitochondria‐GFP labeling was assessed by calculating the percentage of the area within an islet containing GFP fluorescence. After 72 hours of coculture, 0.32% ± 0.06% of the islet area of control islets (normoxia) contained mitochondria‐GFP labeling, which was increased to 1.22% ± 0.21% in hypoxia pre‐exposed islets (Figure 6E). Mitochondria‐GFP was evident within phalloidin‐positive structures localized to β‐cells (Figure 6F, arrowhead, and Figure 6G), consistent with mitochondrial transfer through TNTs to hypoxic mouse islets.

## 4 DISCUSSION

In other tissues, mitochondrial transfer from MSCs is associated with the rescue of metabolic viability in recipient cells which have been subjected to ischemic and inflammatory stresses^13^, ^14^, ^15^, ^16^, ^17^ but, to our knowledge, this is the first report of mitochondria transfer into insulin‐secreting β‐cells in mouse and human islets. β‐cells are metabolically active and use mitochondrial ATP generation to couple elevations in circulating glucose to β‐cell depolarization and the exocytotic release of insulin.^32^ Islet mitochondria are particularly vulnerable to hypoxic stresses during the isolation, purification, and in vitro culture of islets, and impaired mitochondrial mass and/or function results in defective insulin secretion and reduced β‐cell survival.^33^ Accordingly, islet mitochondrial OCR is a key predictor of islet transplantation outcome.^18^, ^19^, ^20^ Numerous studies have demonstrated that MSCs improve β‐cell function in vitro and in vivo and our observations suggest that the transfer of functional mitochondria may be an important mechanism underlying these beneficial effects.

Thus, we consistently observed MSC‐derived mitochondria‐GFP localized within >80% of human β‐cells located in the outer 25 μm of each islet and at the region of direct contact with cocultured human MSCs. Mitochondrial‐GFP was also observed penetrating as far as 40 μm into the 3D islet structure. The average islet diameter is approximately 150 μm,^34^ so our observations suggest that up to 30% of β‐cells are recipient to MSC‐derived mitochondria, sufficient to induce a functional phenotype in the intact islets. The transfer of mitochondria to human β‐cells increased during the first two days of coculture with no further increase thereafter, consistent with our previous reports that MSC coculture induces a significant potentiation of insulin secretion after 2 and 3 days, but not prior to this.^5^, ^11^

Our measurements of mitochondria transfer from mouse MSCs to cocultured mouse islets showed less extensive transfer than that seen in our studies using human cells. This is unlikely to reflect an inability of mouse MSCs to transfer mitochondria. MSCs are heterogeneous in their expression of soluble bioactive molecules, and their functional characteristics, including mitochondrial transfer capacity,^30^ can vary depending upon tissue source, species, and passage number.^35^ However, our measurements consistently demonstrated less extensive mitochondrial‐GFP labeling in mouse islets cocultured with mouse BM‐MSCs, mouse adipose MSCs, and human adipose MSCs when compared to human islets, suggesting that the species variation was not due to the superior functional capacity of human adipose MSCs to donate their mitochondria to neighboring islet cells. The most likely explanation for the more extensive mitochondrial transfer to human islets is that the level of cellular stress in isolated human islets is much greater than that of isolated mouse islets, partly because of the differences in the isolation processes and partly because of donor differences. Human islets experience a prolonged cold ischaemia time during the isolation process and they express characteristic hypoxia inducible factor‐1α regulated genes, with a gene expression profile following culture under normoxic conditions (20% O_2_) which resembles that of mouse islets exposed to hypoxia (1% O_2_).^31^ Thus, human islets are subjected to more extreme cellular stressors than mouse islets which may induce “danger signals”^36^ for MSCs, initiating the donation of MSC‐derived mitochondria to human β‐cells. Our observations of increased MSC‐mediated mitochondria transfer to hypoxia‐exposed mouse islets are consistent with this and suggest that MSCs are most effective in supporting the secretory function of compromised β‐cells. Transfer of mitochondria from MSCs into β‐cells may explain the observed effects of MSC coculture to increase islet OCR in response to elevated glucose. The increased flux of oxidative phosphorylation may, in turn, explain the effects of MSCs to enhance GSIS which we have reported previously,^3^, ^4^, ^5^, ^8^, ^11^ and confirmed in the current study.

Differences in islet donors may also influence experimental outcomes. Human islets are isolated from pancreases harvested from heart‐beating, brain‐dead donors and factors such as age, BMI, and duration of brain death have been shown to impact upon human islet isolation success and on islet function in vitro.^37^, ^38^ In contrast, mouse islets are isolated rapidly from healthy, lean, genetically homogenous, young animals. MSCs are reported to transfer mitochondria to cells deficient in mtDNA but not to otherwise healthy cells,^16^, ^39^ and previous studies have shown an age‐related decline in mtDNA copy number in isolated human islets.^40^ Most of our human islet donors were in middle age, in contrast to the relatively young 8‐ to 12‐week‐old mouse donors used in most islet studies. Differences in islet architecture^41^, ^42^ and differential expression of cell adhesion molecules may also contribute to differences in mitochondria transfer. For example, transfer of MSC‐derived mitochondria to lipopolysaccharide‐injured alveolar epithelial cells was dependent upon connexin 34 (Cx43)‐mediated alveolar attachment^15^ and human and mouse islets differ in their expression of adhesion molecules and gap junctional complex (GJC) components, including connexins.^43^ Our imaging studies have demonstrated the presence of mitochondria‐GFP microvesicles predominantly where MSCs are in direct contact with human islets, suggesting that islet‐MSC contact is required for the transfer of mitochondria and subsequent improvements in islet insulin secretory function. In accordance, our previous studies have demonstrated that indirect transwell MSC‐islet coculture does not improve GSIS, in contrast to the robust improvements we have consistently observed using direct contact coculture of islets with MSCs. 3, 4, 5 Several mechanisms of mitochondria transfer from MSCs to injured tissues have been proposed, including microvesicles, TNTs,^14^, ^26^, ^44^ mitochondrial extrusion, and cytoplasmic fusion with the recipient cells (reviewed in Reference 36). In a mouse model of acute lung injury, mitochondrial donation to alveolar epithelial cells was dependent on the stabilization of cell: cell adhesion via the establishment of Cx43‐containing GJCs and subsequent formation of mitochondria‐transferring TNTs.^15^ Mitochondria‐containing microvesicles were shown to bud from both the ends of TNTs and from the MSC cell body, with engulfment of some of the microvesicles by the epithelial cells. We observed extensive MSC‐derived mitochondrial‐GFP labeling 25‐40 μm (3‐4 islet cell layers) into the 3D human islet architecture, consistent with the notion of organelle transfer through TNTs,^29^ which have lengths of several cell diameters.^45^ We also detected TNT‐like structures, composed of F‐actin, spanning between the MSCs and neighboring β‐cells. MSCs shed a diverse population of EVs, including mitochondria‐containing microvesicles (0.1‐1 μm in diameter).^28^ MSC‐derived EVs recapitulate the capacity of MSCs to transfer their mitochondria to recipient cells in models of lung injury^28^ and the transfer of partially depolarized mitochondria from MSCs experiencing oxidative stress to cocultured macrophages enhances MSC survival whilst restoring mitochondrial bioenergetics in the recipient cells.^46^ Together, the vesicular morphology of the mitochondrial‐GFP labeling within human β‐cells and the depth of penetration of the mitochondria transfer into the 3D islet structure are consistent with a mechanism involving both TNTs and microvesicles, as described previously.^15^

## 5 CONCLUSION

In conclusion, MSCs transfer mitochondria to islet β‐cells during in vitro coculture, and this correlates with increased β‐cell mitochondrial oxygen consumption and enhanced glucose‐induced insulin secretion. Mitochondrial transfer from human MSCs to human islets is more extensive than from mouse MSCs to mouse islets, most likely because isolated human islets are exposed to more extreme cellular stressors which initiate mitochondria transfer to human islets. Ensuring optimal β‐cell mitochondrial mass and bioenergetics through MSC‐mediated mitochondria transfer therefore offers a novel strategy for improving the outcomes of clinical islet transplantation as a therapy for T1D.

## CONFLICT OF INTEREST

C.L.R. declared Fellowship Research Grant. P.M.J. declared Grant funding from Diabetes UK (research charity). The other authors declared no potential conflicts of interest.

## AUTHOR CONTRIBUTIONS

C.L.R.: conception and design, financial support, collection and assembly of data, data analysis and interpretation, manuscript writing, final approval of manuscript; E.L.H.: conception and design, financial support, collection and assembly of data, data analysis and interpretation, manuscript writing; A.C.: collection and assembly of data, data analysis and interpretation, manuscript writing; A.N.M.: conception and design, manuscript writing; A.J.F.K.: conception and design, collection and assembly of data, manuscript writing; P.M.J.: financial support, data analysis and interpretation, manuscript writing, final approval of manuscript.

## Supporting information


**Supplementary Table 1** Human islet donor characteristics

## Data Availability

The data that support the findings of this study are available from the corresponding author upon reasonable request.
