# Supplementary material for: Optimizing beta cell function through mesenchymal stromal cell‐mediated mitochondria transfer
Source: Stem Cells. 2020 Jan 8;38(4):574–84. doi: 10.1002/stem.3134 (PMC7187381; doi:10.1002/stem.3134)
Supplement: Supplementary file 1 — Supplementary Table 1 Human islet donor characteristics [file STEM-38-574-s001.docx]

**Supplementary Table 1.** Human islet donor characteristics

| Donor Age (years) | Gender | Body Mass Index (kg/m^2^) | Viability  (%) | Purity  (%) |
| --- | --- | --- | --- | --- |
| 60 | Male | 32.3 | 80 | 90 |
| 23 | Female | 19.8 | 85 | 90 |
| 52 | Female | 23.4 | 80 | 75 |
| 43 | Female | 30.0 | 75 | 65 |
| 33 | Female | 39.5 | 90 | 80 |
| 60 | Male | 31.0 | 80 | 90 |
